# One or two things we know about concept drift—a survey on monitoring in evolving environments. Part B: locating and explaining concept drift

**DOI:** 10.3389/frai.2024.1330258

**Published:** 2024-07-19

**Authors:** Fabian Hinder, Valerie Vaquet, Barbara Hammer

**Affiliations:** Faculty of Technology, Bielefeld University, Bielefeld, North Rhine-Westphalia, Germany

**Keywords:** concept drift, drift detection, drift localization, drift explanation, monitoring, explainability, survey

## Abstract

In an increasing number of industrial and technical processes, machine learning-based systems are being entrusted with supervision tasks. While they have been successfully utilized in many application areas, they frequently are not able to generalize to changes in the observed data, which environmental changes or degrading sensors might cause. These changes, commonly referred to as concept drift can trigger malfunctions in the used solutions which are safety-critical in many cases. Thus, detecting and analyzing concept drift is a crucial step when building reliable and robust machine learning-driven solutions. In this work, we consider the setting of unsupervised data streams which is highly relevant for different monitoring and anomaly detection scenarios. In particular, we focus on the tasks of localizing and explaining concept drift which are crucial to enable human operators to take appropriate action. Next to providing precise mathematical definitions of the problem of concept drift localization, we survey the body of literature on this topic. By performing standardized experiments on parametric artificial datasets we provide a direct comparison of different strategies. Thereby, we can systematically analyze the properties of different schemes and suggest first guidelines for practical applications. Finally, we explore the emerging topic of explaining concept drift.

## 1 Introduction

The environment around us is constantly changing. While humans are capable of navigating an ever-changing environment, these changes pose challenges to many automated systems (Ditzler et al., 2015). Considering monitoring and control tasks, e.g., in critical infrastructure (Vrachimis et al., 2022), manufacturing (Chen and Boning, 2017), and quality control (Gabbar et al., 2023), in order to work reliably, automatized processes and supervision algorithms need to be able to detect, react, and adapt to changes (Reppa et al., 2016).

Formally, these changes can be described as *concept drift* (or drift for short)—a change in the data generating distribution (Gama et al., 2014). They can be caused by changes in the observed process, the environment, or the sensors acquiring the data. When monitoring a system, for instance in manufacturing or quality control, it is crucial to detect changes in the observed process as this might indicate faulty productions or general malfunctions. In automated processes, on the other hand, it is important to detect changes in the sensors and the environment to take appropriate actions, e.g., replacing a faulty sensor or adapting the system processing the collected data to a changed scenario (Gama et al., 2004, 2014; Gonçalves et al., 2014).

Most often, we consider drift in *stream setups* (Ditzler et al., 2015; Lu et al., 2018), where the underlying data distribution changes. This requires models to adapt or to inform a human operator to take appropriate action. This is closely related to concept evolution in *continual learning* (Delange et al., 2021), commonly discussed in deep learning, where concepts might appear or vanish. Besides, data streams might suffer from temporarily extreme class imbalances or the availability of features might change over time. This can trigger the problem of so-called catastrophic forgetting where a model cannot properly process samples of a class after updating anymore. Drift is not limited to data streams but can also occur in *time-series* (Aminikhanghahi and Cook, 2017) where the single observations are highly interdependent (Esling and Agon, 2012). Here, drift mainly occurs in the form of trends. Commonly its absence is referred to as *stationarity*.

Besides the settings where the data samples are collected over time, considering manufacturing and quality control, frequently data is collected at different locations and processed in the scheme of *federated learning* (Zhang et al., 2021). Here, instead of gathering all the data at a global server processing is done locally and the results are then combined to get an overarching model at the server. Similar to stream learning, in this setting it is necessary to account for differences or drift in the data collected at different locations to obtain a robust global model (Liu et al., 2020). Finally, drift must be considered when performing *transfer learning*, a strategy in deep learning (Pan and Yang, 2010). The basic idea is to deal with limited data by pre-training a model on a similar task using a more extensive dataset and later fine-tuning it to the goal task using a limited dataset at hand. In this work, we will focus on data streams only. However, many of the discussed strategies can be directly applied to the previously named tasks.

Considering processing drifting data streams there are two main groups of tasks. One is to keep a valid model performing some predictive task on the data, e.g., classifying a product into different categories or estimating a property of interest (*online or stream learning*), another goal is to *monitor a system for anomalous behavior* to react appropriately. In this work, we will not consider online learning, as many surveys are providing a good overview of this task (Ditzler et al., 2015; Losing et al., 2018; Lu et al., 2018) and well-established toolboxes exist (Bifet et al., 2010; Montiel et al., 2018, 2021). Instead, we will focus on the monitoring scenario, which is very important in many different settings where drift is expected due to the use of sensor devices or sensitivity to changes in the environment. In this paper, we will focus on methods that help human operators understand drift and related phenomena. As such are not well addressed by loss-based approaches we will focus on distribution-based or unsupervised methods. The precise reasoning for this is provided in Section 2.2. We provide a formal mathematical definition of the main problems, concepts, and notions and a survey of how far these are addressed by current technologies. Moreover, we also have a look at the in-depth analysis techniques like drift localization in data space and the problem of drift explanation.

The task of monitoring is to observe a system and to provide all the information necessary to enable human operators or automatized downstream tasks to take actions that ensure that the system runs properly. Which information is required depends on the specific task (Goldenberg and Webb, 2019; Verma, 2021). However, generally, it can be summarized by addressing the following questions about the drift (Lu et al., 2018):

The first question in every setting concerns *whether (and when)* drift occurs. The task of determining whether or not there is drift during a given time period is called *drift detection* (Gama et al., 2014). In case a drift is detected, additional questions need to be raised to appropriately react to the change in the data distribution. A survey explicitly targeting unsupervised drift detection is provided as the precursor part A (Hinder et al., 2024a) to this paper. Although this paper is self-contained, we suggest the interested reader to consult part A as an informative prior read to ensure that all concepts from the field of drift detection are introduced in depth.

A second question of interest might concern the severity of the drift, as this might influence which kind of action needs to be taken. Usually *drift quantification* (Lu et al., 2018) can be realized as an intermediate step in drift detection: many methods for drift detection estimate the rate of change by some kind of metric and trigger an alarm if those changes exceed a threshold.

To take appropriate action, it is important to pinpoint the drift more precisely. While drift detection and quantification deal with the when by assigning drift-related information to the time component, i.e., finding change points, or determining the rate of change, *drift localization* (and *segmentation*) (Lu et al., 2018) focus on the *where* and assign drift-related information to the data points or space. Consider for example quality control. There might already be an algorithm in place screening the data for known anomalies. However, in case new anomalies in the product occur it is required to detect those to analyze whether some action is required, e.g., discarding the item. In this case, it is crucial to identify the anomalous items, i.e., the drifting data samples, for further analysis. We will focus on drift localization in Section 3.

In some settings answering the discussed questions is not sufficient. In some systems, a malfunction, i.e., the drift, causes a change in several features in all data points collected after the drift event. For example, this might be the case if a sensor is degrading and thus yielding changed measurements. In this case, only using drift localization does not provide much information about what happened in a comprehendible way. Instead, we need more detailed information of *what* exactly happened and *how* it can be described. Providing these detailed, complete, and human-understandable descriptions of ongoing drift is referred to as *drift explanation* (Hinder et al., 2023a). Such methods are designed to support human operators by providing relevant information on monitoring and adaptation processes. This is relevant as the complexity of drift can easily surpass the level of information which is provided by change points or the estimate of the rate of change. Indeed, drift can manifest in a change in the correlation of several features alone, making it nearly impossible for humans to observe without machine aid. In a sense, drift explanations can be seen as the explainable AI (XAI) counterpart for drift detection: while usual XAI explains why a model makes a decision (Gunning et al., 2019; Molnar, 2020), drift explanations provide an explanation of why a drift detector alerts for drift. This commonly makes use of various techniques, including classical XAI. We provide an overview of the most advanced drift explanation schemes in Section 4.

This paper is structured as follows. First, we provide a formalization of concept drift (Section 2.1), and position this paper both in the body of related work in the intersection of the stream setup and supervised and unsupervised approaches (Section 2.2). Afterward, we focus on drift localization (Section 3): We first formalize the task and provide a general scheme most approaches realize. We then discuss a number of methods and approaches from the literature and finally analyze the strategies concerning drift and stream-specific criteria. Before concluding this survey (Section 5), we discuss drift explanation by highlighting some of the most advanced and interesting contributions (Section 4).

## 2 Concept drift—defining the setup

Before we look at drift localization and drift explanations in detail, in this section, we formally define drift and discuss different setups for working with it.

### 2.1 A formal model of concept drift

In the classical setup of machine learning, one assumes that the distribution at training, testing, and application time is always the same, i.e., we assume that the data generating distribution D is time-invariant. In this case, a sample of size *n* is a collection of i.i.d. random variables $X_{1},\ldots ,X_{n}\sim D$.

As discussed before, the assumption of time-invariant distributions is violated in many real-world applications, in particular, when learning on data streams. To resolve this issue from a purely formal point of view, we incorporate time into our considerations by allowing every point to follow a potentially different distribution $X_{i}\sim D_{t_{i}}$ that depends on the time point *t*_*i*_ of observation. As it is unlikely to observe two samples at the same time, i.e., *t*_*i*_ ≠ *t*_*j*_ for all *i* ≠ *j*, it is common to simply write $D_{i}$ instead of $D_{t_{i}}$ (Gama et al., 2014). This relates to the classical setup if all *X*_*i*_ follow the same distribution, i.e., $D_{i}=D_{j}$ holds for all *i, j*. One speaks of *concept drift* if this assumption is violated, i.e., there exists *i, j* such that $D_{i}\neq D_{j}$ (Gama et al., 2014).

However, as pointed out by Hinder et al. (2020) this definition of concept drift depends on the used sample and not on the underlying process. In particular, when taking two samples from the same data source over the same period of time using different sampling frequencies, we might end up with one sample having concept drift while the other does not. This makes understanding concept drift a hard problem. To deal with the issue, it was suggested to take the statistical properties of time into account (Hinder et al., 2020). To do so we consider a model of time T rather than a mere index set. We assume that there is a distribution *P*_*T*_ on T that describes the likelihood of observing a sample at time *t*, and a collection of distributions $D_{t}$ for all t∈T albeit, in practice, only a finite number of time points is observed. Together *P*_*T*_ and $D_{t}$ form what we refer to as a *distribution process* (in the literature this is also referred to as drift process).

Definition 1. Let T=[0,1] and $X={\mathbb{R}}^{d}$. A *(post hoc) distribution process* $\left(D_{t},P_{T}\right)$ from the *time domain* T to the *data space* X is a probability measure *P*_*T*_ on T together with a Markov kernel $D_{t}$ from T to X, i.e., for all t∈T, $D_{t}$ is a probability measure on X and for all measurable A⊂X the map $t\mapsto D_{t}\left(A\right)$ is measurable. We will just write $D_{t}$ instead of $\left(D_{t},P_{T}\right)$ if this does not lead to confusion.^1^

Distribution processes are designed to model *data streams*. In this case, one usually assumes that the observations are independent but may follow different distributions. This setup is not to be confused with *time series* or *stochastic processes* which can be seen as randomly sampling a function from time to data. In particular, this allows the observations to depend on each other. Although both setups can be used to describe the same sequence of data points, there are several subtitle differences in the underlying mathematics and the interpretation (Hinder et al., 2024b). For example, a temperature measurement is a stochastic process as there is only one value for each point in time and the values of successive measurements depend on each other, a stream of ballots on the other hand would be more like a distribution process because the single votes are more or less independent and we are particularly interested in the overall distribution.

Given a distribution process, we can derive two distributions: By adding a time-stamp to every sample the data follows what we will refer to as the holistic distribution D. By collecting all samples observed during a certain time window W⊂T the data follows the mean distribution $D_{W}$. Formally the distributions are given by the following:

Definition 2. Let $\left(D_{t},P_{T}\right)$ be a distribution process from T to X. We refer to the distribution D on X×T which is uniquely determined^2^ by the property $D\left(A\times W\right)=\int_{W}D_{t}\left(A\right)\text{d}P_{T}\left(t\right)$ for all A⊂X,W⊂T as the *holistic distribution* of $D_{t}$. Furthermore, we call a *P*_*T*_ non-null set W⊂T a *time-window* and denote by $D_{W}\left(A\right)=\int_{W}D_{t}\left(A\right)\text{d}P_{T}\left(t|W\right)=D\left(A\times W|X\times W\right)$ the *mean distribution* during *W*.

A benefit of distribution processes is that they allow sampling data. This is in stark contrast to the sample-based setup (Gama et al., 2014), as we cannot create new data points from old ones. Drawing new data from a distribution process can be done in two ways. One option is to draw i.i.d. samples from the holistic distribution D. These samples are dated data points (*X, T*) that can be obtained by the following procedure: First draw the time of observing *X*, i.e., *T* ~ *P*_*T*_, and then draw *X* according to $D_{t}$ assuming *T* = *t*, i.e., $X|\left[T=t\right]\sim D_{t}$. Another sampling method relating to common practical approaches is to take i.i.d. samples from $D_{W}$ for some time window *W*. Notice that a collection of observations that are collected during a time window *W* according to D are exactly distributed according to $D_{W}$. Hence, both ways to sample are formal descriptions of practical relevant procedures to obtain data over time.

We derive a definition for drift in the setup of distribution processes from the definition above: a distribution process has drift if the change of deriving a sample from it that has drift in the sense of Gama et al. (2014) is larger 0, i.e., for a sample *X*_1_, *X*_2_, …  there are *i, j* such that


$$\begin{array}{l} {\mathbb{P}}_{X_{i}}\overset{\text{def.}X_{i}\;}{=}D_{T_{i}}\neq D_{T_{j}}\overset{\text{def.}X_{j}\;}{=}{\mathbb{P}}_{X_{j}} \end{array}$$

and such a sample is observed with a chance larger than zero. Due to measure theoretical reasons the number of samples actually does not play a role so we can also consider only two samples. Thus, we obtain the following definition:

Definition 3. Let $\left(D_{t},P_{T}\right)$ be a distribution process. We say that $D_{t}$ has *drift* iff


$$\begin{array}{l} {\mathbb{P}}_{T,S\sim P_{T}}\left[D_{T}\neq D_{S}\right]=P_{T}^{2}\left(\left\{\left(t,s\right)\in T^{2}|D_{t}\neq D_{s}\right\}\right)>0. \end{array}$$

Here $P_{T}^{2}$ denotes the product measure of *P*_*T*_ with itself, i.e., the measure on $T^{2}=T\times T$ that is uniquely determined by $P_{T}^{2}\left(W_{1}\times W_{2}\right)=P_{T}\left(W_{1}\right)P_{T}\left(W_{2}\right)$.

One may be wondering why this is different from the existence of s,t∈T with $D_{t}\neq D_{s}$. Formally speaking this has to do with *P*_*T*_ null sets. It might happen that the difference only occurs in such a short amount of time, that we will never see only a single sample drawn from the other distribution and thus we will never be able to observe the drift in the data. It is thus a mere artifact of the formal model, rather than the actual process. Hinder et al. (2020) provide several other, equivalent formalizations which relate to scenarios which have been considered in the literature of concept drift are given: being not equal to a standard distribution, i.e., ${\mathbb{P}}_{T\sim P_{T}}\left[D_{T}\neq P\right]>0$ for all distributions *P* on X; being not equal to the mean distribution, i.e., ${\mathbb{P}}_{T\sim P_{T}}\left[D_{T}\neq D_{T}\right]>0$; different distributions for two time-windows, i.e., $D_{W}\neq D_{W^{\prime }}$ for some $W,W^{\prime }\subset T$. One of the key findings however is the equivalence of drift and data *X* and time *T* being dependent.

Theorem 1. *Let* $\left(D_{t},P_{T}\right)$ *be a distribution process from* T *to* X *and let* (X,T)~D *be distributed according to the holistic distribution. Then* $D_{t}$ *has drift if and only if* *T* ⫫ *X* *are not statistically independent, i.e., there exist* W⊂T *and* A⊂X *such that* ℙ[*T* ∈ *W, X* ∈ *A*] ≠ ℙ[*T* ∈ *W*]ℙ[*X* ∈ *A*].

This concept was pivotal in shaping the development of new methods, e.g., it was used to reduce the problem of drift detection to independence *X* ⫫ *T* testing without the necessity of using two windows (Hinder et al., 2020); it was used to describe the location of drift through temporal homogeneity using conditional independence *X* ⫫ *T* ∣ *L*(*X*) where *L* are the homogeneous components (Hinder et al., 2021b, 2022b); explaining drift was reduced to the explanation of models that estimate *X* ↦ *T* (Hinder et al., 2023a); the position of anomalies in critical infrastructure was identified as those features *X*_*i*_ that have a particularly strong correlation with time *T* (Vaquet et al., 2024a,b).

### 2.2 Concept drift in supervised and unsupervised setups

In the last section, we provided a definition of drift based on the data-generating process. Drift is usually further categorized. A first general distinction is usually drawn based on the way drift manifests itself in time. While the distribution might completely change at time *t* (*abrupt drift*), there are also slower changes occurring over an interval of time. In contrast, in *incremental drift*, the distribution changes smoothly over time. In *gradual drift*, during the period of change, the samples are drawn from both distributions with different probabilities. Finally, in many real-world applications, we expect old distributions to reoccur, for instance, due to seasonalities. This phenomenon is referred to as *reoccurring drift*. Notice that some authors refer to abrupt drift as “concept shift” and only call a continuous change “concept drift”. If not further specified we consider all the aforementioned kinds of drift at once.

Besides, one categorizes drift according to how the distribution changes in the data and label space. Assuming that the data stream consists of labeled-data-pairs (X,Y)∈X×Y, where *Y* is the label, in addition to the changes in the joint distribution of *X* and *Y*, the marginal and conditional distributions are of interest. Usually, a change of the posterior $D_{t}\left(Y|X\right)$ is referred to as *real drift*, and a change of the marginal $D_{t}\left(X\right)$ is referred to as *virtual drift*. Sometimes virtual drift is also called *data drift*, while real drift is referred to as concept drift.

As pointed out by Hinder et al. (2023c), from a statistical point of view, drift in the marginal distribution $D_{t}\left(X\right)$ and the joint distribution $D_{t}\left(X,Y\right)$ can be modeled in a common mathematical framework, although the interpretations are of course different. In particular, in both cases drift is equivalent to the statistical dependency of time *T* and (labeled-) data *X* and (*X, Y*), respectively, if the time-enriched representation (holistic distribution) is considered. Real drift on the other hand is equivalent to conditional statistical dependence of label *Y* and time *T* given data *X*, i.e., *Y* ⫫ *T* ∣ *X* (Hinder et al., 2023c).

Analogous to general machine learning tasks, we can consider *supervised* settings, i.e., those that are concerned with conditional distributions, and *unsupervised* tasks, i.e., those that are concerned with the joint or marginal distributions. While in supervised settings both real and virtual drift might be present, in unsupervised settings only virtual drift has to be considered.

**Figure 1:**
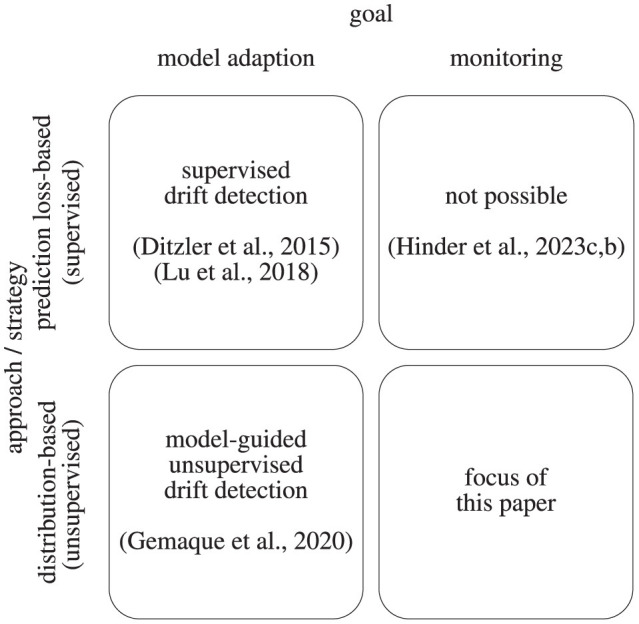
Display of the drift analysis categorization according to the goal and the applied strategy.

As briefly discussed before, there are two main goals when facing drifting data streams. One is to *keep an accurate learning model* even if the data stream is drifting, the other is to accurately detect and describe the drift in the data distribution (*monitoring*) for example to initiate adequate actions. These two goals intersect with two overarching approaches concerning the discussed settings. While in a supervised setting of drift detection, one is usually relying on analyzing *model-losses* as a proxy, i.e., how well a specific model can reconstruct the data or perform a prediction or forecasting task, in unsupervised settings one considers the *data distribution* directly. Structuring approaches according to these dimensions, we obtain a categorization shown in Figure 1.

When it comes to automated model adaption, relying on model loss as an indicator is a reasonable choice and a considerable amount of works and surveys address this issue (Ditzler et al., 2015; Losing et al., 2018; Lu et al., 2018). Much fewer works focus on unsupervised distribution-based drift detection which has been summarized by Gemaque et al. (2020) for model adaption and by Hinder et al. (2024a) for the monitoring setup.

In contrast to detection, in this work, we will focus on localizing, analyzing, and explaining drift. That is, we focus on the monitoring setup with the goal of obtaining knowledge on the drift. This knowledge might be used in further downstream tasks that usually involve human operators, e.g., provoke further analysis of the data or construction works that may benefit from the additional knowledge on the problem at hand (Vaquet et al., 2024a,b).

It is important to notice that the limitations on performing drift detection based on prediction losses translate to this setup. As discussed by Hinder et al. (2023c,b) the connection between model loss, model adaption, and drift is rather vague and heavily depends on the used model class and the precise setup, e.g., Hinder et al. (2023c) give constructive proofs for the fact that there is always purely virtual drift that affects the decision boundary of the optimal model and real drift that neither affects the decision boundary nor the loss of the optimal model under the assumption of finite VC-dimension. It is thus unclear what information is extracted by a loss-based approach if any at all. Therefore, relying on a purely loss-informed approach for explaining drift is usually not reasonable. Hence, we will focus on unsupervised approaches.

However, notice that unsupervised approaches for analyzing drift are also interesting when analyzing drift with the goal of model adaption in mind. Similar to the drift detection case they provide a full picture of the drift while loss-based approaches tend to filter out the information that is not covered by the loss (Hinder et al., 2023c). Since which information is filtered out is independent of whether that information is relevant to the adaption that needs to take place, considering unsupervised explanations can be beneficial. There exist surveys on unsupervised drift detection for model adaption such as Aminikhanghahi and Cook (2017), Lu et al. (2018), and Gemaque et al. (2020) which mention unsupervised drift detection and analysis to some extent but do not provide a broad overview in a more in-depth analysis of the drift as is our goal. To the best of our knowledge, no structured survey has been conducted on drift analysis for this particular task.

## 3 Drift localization and segmentation

Solely detecting and determining the time point of the drift is not sufficient in many monitoring settings. In order to take appropriate action, more questions concerning the drift have to be answered. In this section, we focus on the *where*—our goal is to identify the drifting data points or components in data space.

### 3.1 Problem setup and challenges

**Figure 2:**
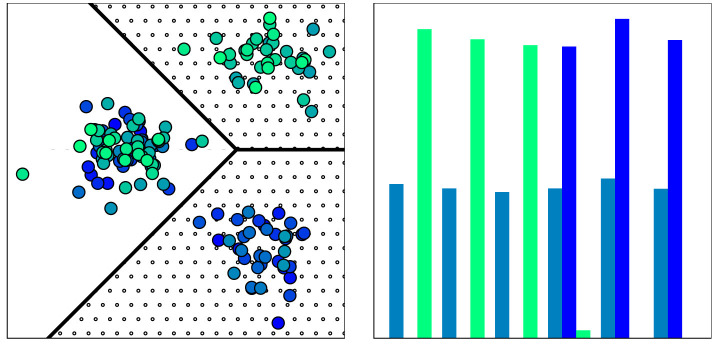
Visualization of distribution consisting of three segments (optimal drift segmentation; borders given by black lines). The two segments on the right form the minimal drift locus (dotted area). **(Left)** Sample drawn from distribution color indicates time point of observation, evolving from dark blue to light green, **(right)** Time point distribution per segment; green and blue bars show lower and upper segment on the right, petrol bars show left segment.

The task of determining where in data space the detected drift manifests is referred to as *drift localization*. Informally, the problem of drift localization can be expressed as “finding those regions in the data space that are affected by the drift” (Dasu et al., 2006; Lu et al., 2018). We have illustrated this in Figure 2, the dotted area is the area of interest. A slightly different angle on this question would be investigating “whether or not a given data point is affected by the drift” (Liu et al., 2017; Hinder et al., 2022b). Both questions are relevant in practical applications. If we know which parts of the data space are affected by the drift, we know which analysis has to be redone. On the other hand, if we know which data points are affected by the drift we can update our dataset more efficiently, i.e., we do not need to discard all old data points but only the affected ones.

Both questions can be raised interchangeably: if we know which parts of the data space are affected by drift, we can mark all data points therein as drifting. If, on the other hand, we know for every data point whether or not it is drifting, we can mark the corresponding parts of the data space as drifting. However, in practical applications, identifying the drifting samples is usually more feasible. In particular, we can consider it as a statistical test with the *H*_0_ hypothesis “The data point *x* is not affected by drift”.

To summarize, we want to separate those parts of the dataset/space where the drift manifests from those irrelevant to the drift. This is challenging as the definition of drift is non-local in the sense that it makes no statement about the inner workings of the distribution process. Rather, it simply states that there is some kind of difference in the distributions over time. Yet, from a mathematical point of view, there is no obvious way to talk about the behavior locally, i.e., at a single point. Thus, before we can work on a solution for the task, we first need to specify what we actually mean. For this purpose, we will make use of the formalization of drift localization presented by Hinder et al. (2022b).

When discussing drift in the unsupervised setup one usually imagines something like a Gaussian moving through space, i.e., $X={\mathbb{R}}^{d}$ and $D_{t}=N\left(\mu_{t},\sigma \right)$ where $\mu_{t}:T\to {\mathbb{R}}^{d}$ is the moving mean of the Gaussian. However, as is well known Gaussians span the entire space and are thus not suited for analyzing the local properties of the drift. Instead, we suggest to approximate the distribution process using a mixture model of uniform distributions on a grid: denote by $L_{i_{1},\ldots ,i_{d}}^{\left(n\right)}=\left[i_{1}/2^{n},\left(i_{1}+1\right)/2^{n}\right)\times \cdots \times \left[i_{d}/2^{n},\left(i_{d}+1\right)/2^{n}\right)$ the grid cell starting at $\left(i_{1},\ldots ,i_{d}\right)/2^{n}$ with length 1/2^*n*^. A grid-based approximation is then given by


$$\begin{array}{l} {\hat{D}}_{t}^{\left(n\right)}=\underset{i_{1},\ldots ,i_{d}\in \mathbb{Z}}{\sum }\lambda_{i_{1},\ldots ,i_{d}}\left(t\right)U\left(\;L_{i_{1},\ldots ,i_{d}}^{\left(n\right)}\;\right) \end{array}$$

where $\lambda_{i_{1},\ldots ,i_{d}}^{\left(n\right)}\left(t\right)=D_{t}\left(\;L_{i_{1},\ldots ,i_{d}}^{\left(n\right)}\;\right)$ are the time-dependent weights assigning with which probability a grid cell will be present in the data at the considered time.

**Figure 3:**
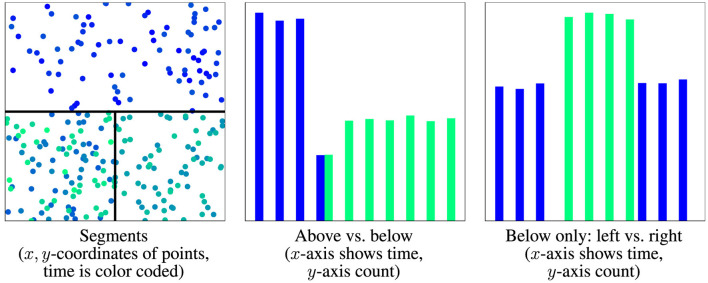
Visualization of distribution with three segments, all having drift. **(Left)** Optimal drift segmentation, time is color coded, black lines mark segment border. **(Middle/right)** Time point distribution of segments. First drift moves distribution from upper halve to left below, second from left below to right below, and third from right below to left below. The upper half is a drift segment (no drift inside), and the lower half is not as the distribution moves from left to right and back.

Observe that ${\hat{D}}_{t}^{\left(n\right)}$ approximates $D_{t}$ in the weak sense and that it has drift if and only if at least one weight function λ_*i*_1_, …, *i*_*d*__(*t*) is not constant. If $D_{t}$ has no drift, then neither does ${\hat{D}}_{t}^{\left(n\right)}$ the converse however does only hold for sufficiently large *n*. In more detail, by applying a local approximation, the drift that “moves” probability between the cells is captured as a change of the weights $\lambda_{i_{1},\ldots ,i_{d}}^{\left(n\right)}\left(t\right)$, while the drift happening inside a cell is not captured by ${\hat{D}}_{t}^{\left(n\right)}$. For example, see Figure 3 for an illustration or consider $D_{t}=\delta_{t/2^{m}}$ for X=ℝ,T=[0,1] then ${\hat{D}}_{t}^{\left(n\right)}$ has drift only if *n* > *m*.

In some cases, we can choose *n* such that there is no drift inside any of the cells $L_{i_{1},\ldots ,i_{d}}^{\left(n\right)}$. In this case, the entire drift is encoded in the weights $\lambda_{i_{1},\ldots ,i_{d}}^{\left(n\right)}\left(t\right)$. The drifting behavior inside the cells can thus be considered homogeneous. Note that ${\hat{D}}_{t}^{\left(n\right)}=D_{t}$ is not necessary but it suffices that the precise location of a point provides no more information on the drift than the cell containing it. We will refer to such sets L⊂X as *drift segments*. Of course, other choices than grid cells are also possible. Assuming we can cover the data space by disjoint segments *L*_1_, …, *L*_*n*_ then the drift can be fully described by the weight functions $\lambda_{i}\left(t\right)=D_{t}\left(L_{i}\right)$.

In drift detection, virtual classifiers (Kifer et al., 2004) use similar ideas. They are trained to classify the samples in the reference *W*_−_(*t*) and current window *W*_+_(*t*) as -1 and +1, respectively. Classifier performance is then used as a drift score or test statistic. If the classification is perfect, then the sets of all points classified as -1 or 1 form drift segments, although this is not necessary. In Figure 3, the classifier may choose *L* as the upper halve and then observe the discrepancy presented in the middle diagram.

To give a formally sound definition of drift segments we need to define how to obtain a distribution process on a sub-space, i.e., we need a spatial restriction of the kernel $D_{t}$ from X to *L*. Then *L* is homogeneous if the resulting distribution process has no drift. As kernels are not defined point-wise in X we cannot use function restrictions. Instead, we consider the distribution process $\left(D_{t}\left(\cdot |L\right),D\left(\cdot \times L|T\times L\right)\right)$ which is chosen to yield D(·∣T×L) as holistic distribution. Here, we first restrict to *L* by intersecting, i.e., $D_{t}\left(\cdot \cap L\right)$. This is not a Markov kernel as it will usually not be a probability measure. We can normalize by $D_{t}\left(L\right)$ resulting in the conditional $D_{t}\left(\cdot |L\right)$ which is only defined if $D_{t}\left(L\right)\neq 0$. Thus, we also modify the sampling probability to assure $D_{t}\left(L\right)>0$ for almost all observed *t*.

Notice, that the restricted distribution process has desirable properties:

1. It is a distribution process on X concentrated on *L*, i.e., A⊂X\L is assigned Dt(A∣L)=0 *P*_*T*_-a.s.
2. It resembles $D_{t}$, i.e., if $D_{t}$ does admit a density *f*(*x* ∣ *t*) then the restricted process has the density *f*(*x* ∣ *t*)*g*(*t*) for *x* ∈ *L* and 0 otherwise.
3. It is compatible with further restrictions, i.e., if we first restrict to *L* and then to *L*′⊂*L*, then we obtain the same process as by directly restricting to *L*′.

We can now formally define the notion of drift segments:

Definition 4. Let $\left(D_{t},P_{T}\right)$ be a distribution process from T to X. Let L⊂X be a $D_{T}$ non-null set, then the *restriction* of $D_{t}$ onto *L* is the distribution process with kernel $A\mapsto D_{t}\left(A|L\right)=D_{t}\left(A\cap L\right)/D_{t}\left(L\right)$ and time distribution W↦D(W×L∣T×L), where D the holistic distribution of the original distribution process. We refer to $T_{L}=\text{supp}\left(D\left(\cdot \times L\right)\right)$ as the *active time* of *L*, where supp denotes the support of the measure.

A $D_{T}$ non-null set L⊂X is called a *drift segment* if the restriction of $D_{t}$ to *L* has no drift. A drift segment is called maximal iff it is maximal with respect to set inclusion, i.e., if for every *L* ⊂ *L*′ we either have $D_{T}\left(L^{\prime }\backslash L\right)=0$ or the restriction with respect to *L*′ has drift.

A collection of drift segments *L*_*i*_, *i* ∈ ℕ that cover X, i.e., $\cup_{i}L_{i}=X$, is called a *drift segmentation*. If all segments are maximal then the segmentation is *optimal*.

The notion of a maximal drift segment comes from the observation that if *L* is a drift segment, then every subset *L*″⊂*L* is a drift segment, too. Thus, maximality enforces that the segments are of reasonable size.

We will now define drift localization. As it is simpler, we define the drifting region as the complement of the non-drifting region, i.e., which part of the distribution has to be “removed” in order to make the drift disappear. As drift segments are only homogeneous there can still be drift between the segments, e.g., in Figure 3 there are three segments, but every single point in the data space is affected by drift. If we also take the drift between the segments into account we have to add that $t\mapsto D_{t}\left(L\right)$ is constant. Consequently, we have $D\left(W\times L|T\times L\right)=P_{T}\left(W\right)$ so we do not need to take active time into account which then leads to the following definition:

Definition 5. A *drift locus* is a measurable set L⊂X such that $\left(D_{t}\left(\cdot |L^{C}\right),P_{T}\right)$ has no drift and $t\mapsto D_{t}\left(L\right)$ is *P*_*T*_-a.s. constant. A drift locus *L* is *minimal* if it is contained in every other drift locus *L*′ up to a $D_{T}$-null set, i.e., $D_{T}\left(L\backslash L^{\prime }\right)=0$. We refer to the task of finding the minimal drift locus as *drift localization*.

The notion of minimality in the definition is analogous to the maximality drifting segment.

The notion of a minimal drift locus has several desirable properties (Hinder et al., 2022b). Among those is the fact that in all practically relevant cases, there is a unique minimal drift locus so the notion of drift localization makes sense from a theoretical point of view. Furthermore, the minimal drift locus is not empty if and only if there is drift. In the following, we will discuss how to obtain a drift localization given data.

### 3.2 A general scheme for drift localization

**Figure 4:**
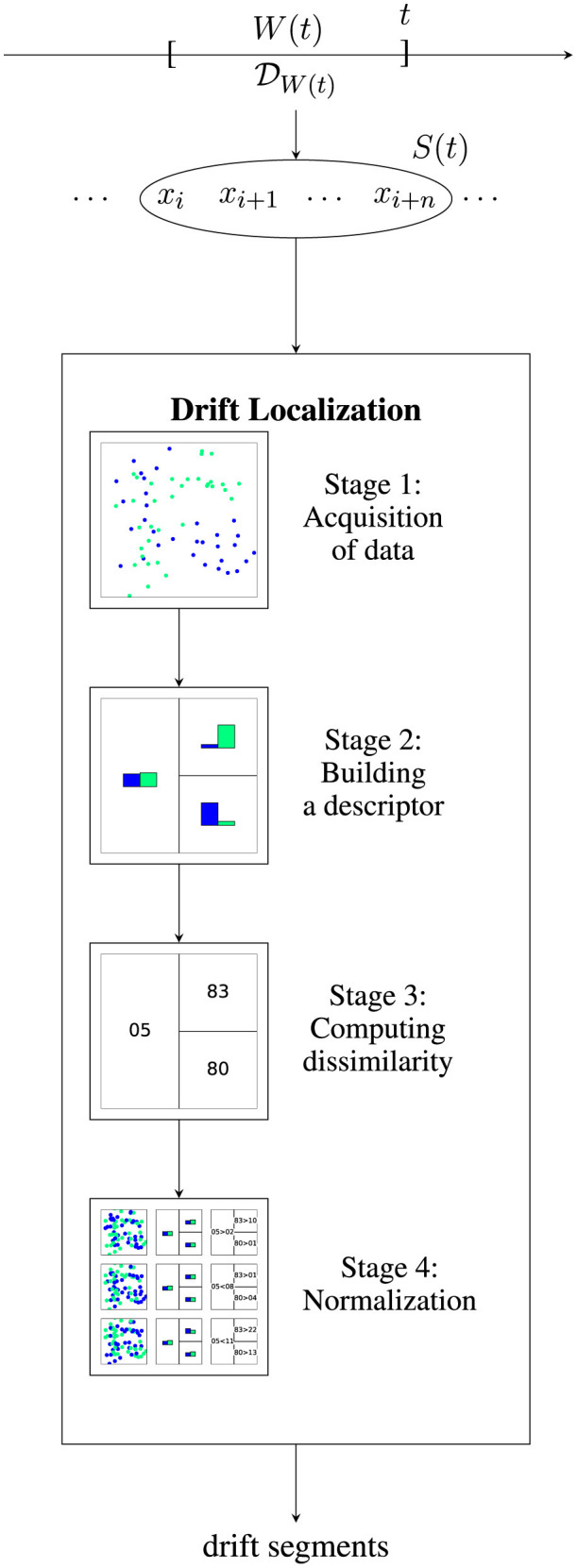
Visualization of drift localization for a data stream. Given a data stream, for each time window *W*(*t*) a distribution $D_{W\left(t\right)}$ generates a sample *S*(*t*). A drift localization algorithm estimates whether *x* ∈ *S*(*t*) is affected by drift or not by performing a four-stage detection scheme. The illustrated algorithm uses two-windows (stage 1), tree-histograms (stage 2), leaf-wise total variation norm (stage 3), and permutation normalization (stage 4).

As discussed before, the goal of drift localization is to investigate where the underlying distribution changes. Drift localization is usually applied in a streaming setting where a stream of data points is arriving over time. At time *t* a sample *S*(*t*) containing some data points which are observed during *W*(*t*) and thus are generated by $D_{W\left(t\right)}$ becomes available. On an algorithmic level, this resembles the way drift detectors work. Lu et al. (2018) provided a four-stage scheme that allows to classify drift detectors in a systematic manner. In the following, we describe an adaption of that scheme for drift localization. We visualized the overall scheme in Figure 4.

#### 3.2.1 Stage 1: acquisition of data

As a first step, we need a strategy for selecting which data points are to be used for further analysis. Most approaches rely on some instantiation of sliding window strategies. There are four main categories that differ in how the reference window is updated, e.g., fixed until an event, growing, or sliding along the stream or implicit as a summary statistic using a model. We refer to Lu et al. (2018) and Hinder et al. (2024a) for a more detailed description. However, as we usually require a large amount of data for the localization task, to the best of our knowledge there are no methods that make use of an implicit reference window. Similar preprocessing steps as for drift detection, such as a deep latent space embedding, are reasonable tools that have been applied successfully in the literature (Hinder et al., 2023a).

#### 3.2.2 Stage 2: building a descriptor

Just as drift detection, drift localization algorithms split the data processing into two steps. First, building a descriptor from data and then analyzing it. In contrast to drift detection, those usually offer a quite direct connection between locations in data space and the structure of the descriptor. Commonly used are binnings, e.g., based on decision trees (Dasu et al., 2006; Hinder et al., 2021b, 2022b), or *k*-neighbor based descriptors (Liu et al., 2017; Hinder et al., 2022b). However, depending on the analysis algorithm nearly arbitrary machine learning models can be used as descriptor (Hinder et al., 2022b).

#### 3.2.3 Stage 3: computing dissimilarity

Based on the descriptor a drift score is computed. In contrast to drift detection, where the score is used to describe the global amount of drift, in drift localization it measures the amount of drift in a region of the data space (Dasu et al., 2006; Hinder et al., 2021b, 2022b) or a single data point (Liu et al., 2017; Hinder et al., 2022b). In particular, for methods that make use of region-wise computations, this can be considered as performing a common drift detection that only takes a small region of the data space into account (Dasu et al., 2006). Many dissimilarities used in drift detection are based on the idea, that there is drift if we can partition the data space in such a way that the number of samples observed before and after the drift differ significantly for the different segments. All drift detection algorithms considered by Hinder et al. (2024a) make use of this idea in one way or another. Particular direct examples are drift detectors based on virtual classifiers (Kifer et al., 2004; Hido et al., 2008).

#### 3.2.4 Stage 4: normalization

Similar to drift detection, the obtained dissimilarities are typically very setup-specific and do not allow a direct conclusion regarding whether or not a certain sample or region is drifting. A common strategy is to make use of some kind of bootstrap- or permutation-based statistical test in order to either find the parameters under the *H*_0_ hypothesis (Liu et al., 2017) or directly compute a *p*-value for the point or region (Dasu et al., 2006; Hinder et al., 2022b).

### 3.3 Approaches

Although several methods for drift detection exist and some allow further analysis of the drift, drift localization in the sense described above is a less popular research question. Most methods that admit such an option either use it as a subroutine of drift detection or allow for it as a mere byproduct (Dasu et al., 2006) rather than an explicit aim for drift analysis technology (Lu et al., 2018). The majority of algorithms that allow localization are based in one way or another on performing drift detection on a local scale (Dasu et al., 2006; Liu et al., 2017; Hinder et al., 2022b), i.e., instead of analyzing the entire dataset at once, only local subspaces are analyzed. This has the drawback that we usually have less data to work with. On the other hand, as we are already local in data space we can make use of far simpler detection schemes.

For example, if we make use of a grid-based binning then the total variation norm is approximated by counting the samples of each distribution per bin, taking the difference of those numbers, and summing up:


$$\begin{array}{l} \hat{∥P-Q∥}=\overset{k}{\underset{i=1}{\sum }}|\frac{1}{m}\overset{m}{\underset{j=1}{\sum }}1\left[X_{j}\in G_{i}\right]-\frac{1}{n}\overset{n}{\underset{j=1}{\sum }}1\left[Y_{j}\in G_{i}\right]|, \end{array}$$

where $G_{1},\ldots ,G_{k}\subset X,\cup_{i=1}^{k}G_{i}=X,G_{i}\cap G_{j}=\emptyset$ for *i* ≠ *j* are the grid-cells, *X*_1_, …, *X*_*m*_ ~ *P*, *Y*_1_, …, *Y*_*n*_ ~ *Q* i.i.d. Similar estimation strategies can be applied to all sorts of distances (Hinder et al., 2022c).

Just as in (global) drift detection, the estimates are usually based on two-window approaches where we first select a split point and then compare the distributions before and after. As in drift detection, this split point can be chosen more or less arbitrarily. However, as the algorithms are usually less robust due to the small sample size, it can be beneficial to first determine the actual split point using a suitable drift detector and then perform the localization. The common next step is to use the descriptor that relates to locations in data space to perform local drift detection. Such descriptors only need to count the number of points in the vicinity of the query point which can be considered as a probabilistic classification task. This idea was further analyzed by Hinder et al. (2022b) giving a theoretical justification for the most approaches available. In particular, it was shown that most probabilistic classifiers can be used for drift localization.

In the following we will consider four exemplary approaches in more detail:

#### 3.3.1 *kdq*-tree

The algorithm is one of the oldest implementations for drift localization (Dasu et al., 2006). It is a two-window approach (stage 1), designed to work with vectorial data only. The main idea is to grow a *kd*-tree-like data structure to obtain a binning (stage 2). More precisely, the trees are obtained by iterating over each dimension in every recursion step and splitting the area right in the middle of said dimension as long as enough data is available. This assures that the volume of every leaf shrinks exponentially with each recursion. Hence, *kdq*-trees do not take the data distribution into account.

Once the tree is grown, it computes the Kullback-Leibler divergence to compare the number of samples coming from each window on every leaf which serves as the drift score (stage 3). This way we obtain a score for every leaf and thus region in the data space. Then a bootstrap is used to compute the threshold (stage 4) which also depends on user-defined parameters. If the score of a leaf exceeds a threshold then the leaf area is considered as drifting.

#### 3.3.2 LDD-DIS

The algorithm (Liu et al., 2017) is a two-window (stage 1), neighbor-based (stage 2) approach that computes a drift score for every data point. It is based on the *Local Drift Degree* which is the ratio of the number of points in the *k*-neighborhood query point categorized by arrival time minus 1 (stage 3). It is 0 if the ratio is even and deviates if there are far more samples from one window than the other. By an application of the central limit theorem, the authors show that under *H*_0_ for large *k* the scores follow a normal distribution. The parameters are estimated using a permutation scheme. This distribution is then used for normalization (stage 4).

Notice that the ratio that forms the heart of the LDD is closely related to the predicted probability of a *k*-neighbor classifier.

#### 3.3.3 Model-based drift localization

A family of algorithms that make explicit use of machine learning models has been introduced by Hinder et al. (2022b). The algorithms can be classified as multiple-window-based approaches (stage 1), i.e., two windows or more. For simplicity and comparability, we will consider the two-window case here.

Very similar to virtual classifiers (Kifer et al., 2004; Hido et al., 2008) and LDD, a probabilistic classifier is trained to predict the window each sample belongs to (stage 2). The drift score, which the authors refer to as informativity, is given by the classifier prediction compared to the prediction of a constant model using the normalized Kullback-Leibler divergence (stage 3). Informativity can thus be interpreted as the information gained by providing the location for predicting time. Informativity takes on values between 0 and 1, with the mean informativity being 0 if and only if there is no drift, and the informativity at one point is larger than 0 if and only if that point belongs to the minimal drift locus. This serves as a theoretical justification for the presented method and other methods like LDD-DIS or *kdq*-trees.

Algorithmically, the authors suggest making use of a permutation test using informativity as test statistic (stage 4). For some models like *k*-nearest neighbor, decision trees, or random forests, the corresponding distribution under *H*_0_ can be computed analytically. Furthermore, due to the supervised training scheme, model parameters can be determined by cross-validation.

#### 3.3.4 Drift segmentation

As stated in Section 3.1, drift localization can be considered as a downstream task of drift segmentation (Hinder et al., 2021b) by checking for each segment whether $D_{t}\left(L\right)$ is constant (stages 3 & 4). Drift segmentation can be approached using the ideas discussed by Hinder et al. (2022b), however, instead of performing probabilistic classification, conditional density estimation is employed (stages 1 & 2), i.e., the model is trained to predict *x*↦ℙ_*T* ∣ *X* = *x*_. This way, it is no longer necessary to choose a split point or several windows. In this sense, drift segmentation relates to “block-based drift detectors” (Hinder et al., 2024a), i.e., approaches that analyze the drift structure of an entire block of data as a whole in contrast to methods that are based on comparing two windows.

Algorithmically, the methods mainly differ in how the descriptor is constructed (stage 2): drift segmentation can be performed using a special decision tree that uses the Kolmogorov–Smirnov test as split criterion to reduce the dependence of data and time (Hinder et al., 2021b). Drift segments are then given by the leaves. This idea was extended to arbitrary segmentation-based multi-regression models (Hinder et al., 2023a) by applying a suited preprocessing to *T* (Izbicki and Lee, 2017; Hinder et al., 2021c). Furthermore, using any multi-regression model, a segmentation can be obtained by applying clustering using a model-informed metric (Hinder et al., 2023a).

### 3.4 An analysis

So far, we discussed drift localization methodologies on a conceptual level. In the remainder of this part, we focus on conducting a numerical analysis and providing guidelines for the practical usage of these methods. For this purpose, we identified three main parameters that describe the data stream and the drift we aim to detect: we investigate the role of the *drift strength*, the influence of drift in *correlating features*, and the *data dimensionality*. We will present and discuss our findings in the remainder of this section.

#### 3.4.1 Experimental setup

*Dataset:* We use the “uniform” dataset from part A (Hinder et al., 2024a): a 2-dimensional, synthetic dataset sampled from a uniform distribution on the unit square. Drift is induced by a shift along the diagonal, i.e., in *x*- and *y*-direction, with different lengths (*intensity*). Besides drift intensity, we also consider the total number of *dimensions* by adding features sampled from uniform random noise, random *rotations* relating to correlated features, and the number of *samples*. We assume that we know the time point of drift and that we are provided with an equal amount of samples before and after the drift.

For the random rotation we first generate the dataset, center at 0, and then apply a random rotation of various strengths across all dimensions, i.e., multiply with λ*O* + (1 − λ)*I* for λ ∈ [0, 1], where *O* is a randomly sampled orthogonal matrix (random rotation) and *I* is the identity matrix (data is axis aligned). We consider all combinations of parameters.

*Method:*^3^ We consider the *kdq*-Tree, LDD-DIS, and Model-Based Drift Localization based on random forests (MB-DL).

*Evaluation:* We perform a sample-based evaluation aiming at identifying for each data point whether or not it is affected by drift. Here, we consider every data point in the overlap of the squares as non-drifting and every other data point as drifting. To evaluate the methods we use the ROC-AUC. It measures how well the obtained drift scores separate the drifting and non-drifting data points. The score is 1 if the largest score assigned to a non-drifting point is smaller than the smallest score assigned to a drifting point, it is 0.5 if the alignment is random. Thus, the ROC-AUC provides a scale-invariant upper bound on the performance of every concrete threshold. Furthermore, in contrast to many other scores like F1 or accuracy, the ROC-AUC is not affected by the expected class imbalance.

#### 3.4.2 Overall results

**Figure 5:**
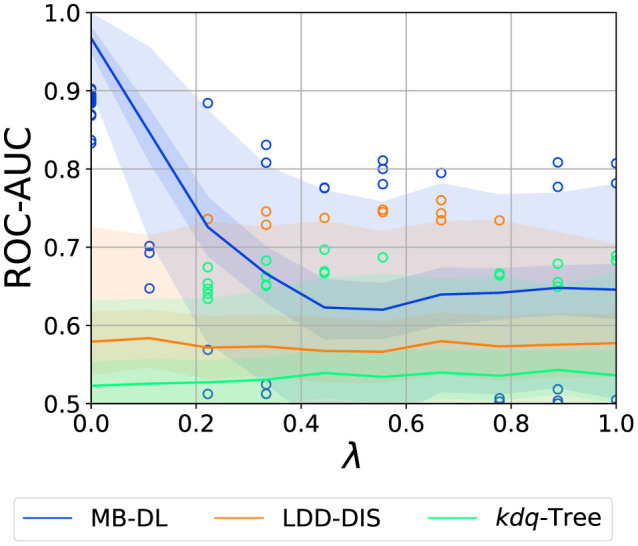
Effect of axis alignment (λ) on localization performance (intensity: 0.05, samples: 750, dimensions: 5). Graphic shows median (line), 25%–75%-quantile (inner area), min–max-quantile (outer area), and outliers (circles).

**Figure 6:**
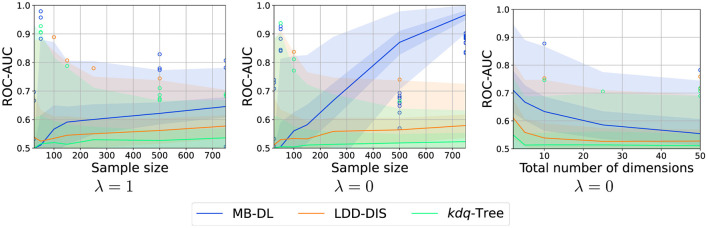
Effect of number of samples and dimensionality on localization performance for various choices of λ (intensity: 0.05, samples: 250, dimensions: 5). Graphic shows median (line), 25%–75%-quantile (inner area), min–max-quantile (outer area), and outliers (circles).

**Figure 7:**
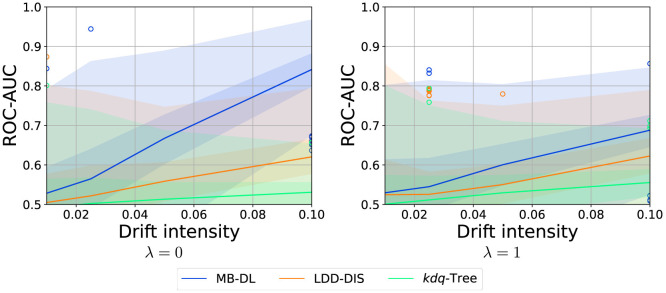
Effect of drift intensity on localization performance for various choices of λ (samples: 250, dimensions: 5). Graphic shows median (line), 25%–75%-quantile (inner area), min–max-quantile (outer area), and outliers (circles).

The overall results of the experiment (see Figures 5–7) show that the problem of drift localization is a comparably hard one and still requires additional research. The overall ranking of the methods in our study places MB-DL at the top, followed by LDD-DIS, and *kdq*-Trees. This is consistent with the findings in the original paper where more complex datasets were analyzed (Hinder et al., 2022b). For all parameters, *kdq*-Trees perform only slightly better than random chance, LDD-DIS barely ever reaches a score of 0.6 or higher. This, together with the very high variance makes the analysis comparably hard.

#### 3.4.3 Axis-alignment

As two out of three methods are tree-based we expect the effect of λ to be significant. As one can see in Figure 5, axis alignment is one of the most crucial parameters for MB-DL, for the other two approaches it is nearly irrelevant. For the MB-DL applied to a window of 150 samples or more, we observe an extreme decline in performance when we switch from λ = 0 (perfectly axis-aligned) to λ = 0.5. After that, the performance stays at a constant, low level. This is to be expected as random forests use axis-aligned splits and thus face problems when classifications require taking correlations into account, as for λ > 0. In the following, we will thus explicitly discuss the cases λ = 0 and λ = 1 separately.

#### 3.4.4 Sample size and dimensions

As is expected all methods profit from larger sample sizes (see Figure 6). However, the increase in performance of *kdq*-Tree is not significant and might be due to random chance. In the case of MB-DL, the increase for λ = 1 is only moderate and comparable to LDD-DIS, for λ = 0 the increase in performance is significant.

Similar results can be found for the number of noise dimensions. While all methods suffer from high dimensionality, MB-DL performs moderately for λ = 0, this is to be expected as the detection scheme is trained in a supervised fashion and thus can perform feature selection in this case. Similar effects cannot be observed for *kdq*-Trees as they do not optimize the tree structure for the problem at hand, LDD-DIS which is not capable of feature selection in the first place, or MB-DL if λ = 1 as in this case feature selection is not possible.

To conclude, drift localization requires a comparably large amount of data and high dimensionality poses a problem in particular if no feature selection is possible. Still, even in this case, model-based approaches might still be the best choice.

#### 3.4.5 Drift intensity

Just as in drift detection, the larger the drift the easier the task becomes. This finding is consistent across all methods as summarized in Figure 7. Furthermore, we see a nearly linear relationship between the drift intensity and the increase of performance. For MB-DL, this is true for λ = 0 and λ = 1, but for the first, the increase is much steeper.

As the split point has a significant impact on the drift intensity (Hinder et al., 2024a) we suggest using a drift detector that estimates the correct split point like ShapeDD (Hinder et al., 2021a) or Kernel Change-point Detection (Harchaoui and Cappé, 2007) to increase localization performance.

### 3.5 Conclusion and guidelines

Investigating the task of drift localization, we provided a formal definition and classified existing approaches according to the four-staged scheme suggested by Lu et al. (2018) for drift detection. Overall, we find that research on drift localization is still very limited with few approaches existing. In our experiments, we only reported good results for a few of the methods. Although we only considered one dataset, the fact that the dataset is very simple suggests that the problem of drift localization is quite hard. Thus, further research is needed.

When applying drift localization methods in practical applications one should pay attention to using a drift detector that estimates the correct change point. Furthermore, one should use as much data as possible as the process appears to be rather data-hungry. Besides, avoiding high dimensional data if possible is essential. In this case, feature selection might offer a good solution (Hinder and Hammer, 2023). Finally, when relying on tree-based methods, it is crucial to design an appropriate preprocessing if the drift inflicts itself in data correlations.

## 4 Drift explanations

As stated in the introduction, addressing drift monitoring involves five main questions: *whether, when, how much, where*, and *what*. The questions on *whether, when*, and *how much* are addressed by drift detection and are discussed in part A (Hinder et al., 2024a). Drift localization (Section 3) addresses the *where*. What remains is the question on *what* happened, i.e., we would like to obtain human understandable descriptions of the drift that enable human operators to perform informed actions. For this, the information provided by drift detection is usually not sufficient and that provided by drift localization and segmentation is not sufficiently condensed to be processed by humans. Therefore, we will focus on a more advanced analysis of the drift in this section.

### 4.1 Problem and setup

The question of *explaining* drift, i.e., describing the potentially complex and high dimensional change of distribution in a human-understandable fashion, is a relevant problem as it enables an inspection of the most prominent characteristics of how and where drift manifests. Hence, it enables human understanding of the change and thus is a key ingredient for informed decision-making by human operators.

In contrast to the problems discussed so far, drift explanations are an inherently ill-defined problem. This is mainly caused by the fact that explanations are inherently ill-posed as can be seen by considering the wide range of different explanation schemes, methods, and frameworks present in the literature. Furthermore, the choice of a suitable explanation is highly domain and problem-specific.

Providing a detailed, complete, and understandable description of ongoing drift requires a large amount of information covering all relevant aspects. This usually surpasses the level of information which is required to select change points or to estimate the rate of change: While drift can be detected based on a single drifting feature, its explanation might need to address the interplay of all drifting features.

This leads us to two general insights on the topic: First, it is not possible to provide a formal definition of what drift explanations are. Second, a large number of different explanation schemes—one for every potential use case—is a desirable state of affairs.

### 4.2 A general scheme for drift explanations

Even though we cannot provide a formal definition of drift explanations, we can still analyze approaches using a similar functional scheme as used for detection and localization (Section 3.2). Here, the normalization stage is usually not required.

#### 4.2.1 Stage 1: acquisition of data

As a first step, we again need a strategy for selecting which data points are to be used for further analysis. Most approaches rely on some instantiation of sliding window strategies. Similar preprocessing steps to drift detection and localization, such as a deep latent space embedding, are reasonable tools that have been applied successfully in the literature (Hinder et al., 2023a).

#### 4.2.2 Stage 2: building a descriptor

Just as in drift detection and localization, drift explanation algorithms split the data processing into two steps building a descriptor from data first and then analyzing it. Similar to drift localization, those are usually chosen with respect to the explanation task at hand. Depending on the desired explanation, a large variety of descriptors is used, but binning approaches are very common (Pratt and Tschapek, 2003; Webb et al., 2016, 2018). However, as pointed out by Hinder et al. (2023a) nearly every machine learning model can be used as descriptors.

#### 4.2.3 Stage 3: computing explanations

In the last stage of the explanation scheme, the descriptor is analyzed. This is comparable to the computation of dissimilarity as a simple quantity derived from the descriptor. Indeed, many methods simply derive numbers such as feature-wise change intensity or change in correlation (Pratt and Tschapek, 2003; Webb et al., 2016, 2018; Wang et al., 2020). Here, a further analysis by means of normalization is not necessary as the data is usually directly presented to and judged by a human operator. However, some more advanced explanation methods are available (Hinder et al., 2023a).

### 4.3 Exemplary cases

While explainability has been a major research interest in recent years (Molnar, 2020; Rohlfing et al., 2021), more complex explanation methods for drift are still limited. Quite a number of approaches aim for the detection and quantification of drift (Lu et al., 2018; Webb et al., 2018), or its visualization (Pratt and Tschapek, 2003; Webb et al., 2017; Wang et al., 2020). Furthermore, several methods focus on feature-wise representations of drift (Pratt and Tschapek, 2003; Webb et al., 2017, 2018; Wang et al., 2020). However, these methods face challenges if high-dimensional data or non-semantic features are dealt with. To the best of our knowledge, there is only one approach that directly targets general concept drift using more complex XAI methods for explaining drift (Hinder et al., 2023a).

In the following, we will group the methods based on the question of whether they focus on feature-wise analysis only, or allow for the application of more complex XAI technologies.

#### 4.3.1 Feature-based drift explanations

Webb et al. (2017, 2018) make use of the (conditional) *drift magnitude* to visualize the intensity and change of correlation of certain features. For sets of features *F, F*′ the drift magnitude is defined as


$$\begin{array}{l} \sigma_{D_{\cdot },l}^{F}\left(s,t\right)=∥D_{W_{l}\left(s\right)}\left(X_{F}\right)-D_{W_{l}\left(t\right)}\left(X_{F}\right){∥}_{\text{TV}}\\ \sigma_{D_{\cdot },l}^{F|F^{\prime }}\left(s,t\right)=\int ∥D_{W_{l}\left(s\right)}\left(X_{F}|X_{F^{\prime }}\right)-D_{W_{l}\left(t\right)}\left(X_{F}|X_{F^{\prime }}\right){∥}_{\text{TV}}\text{d}D_{W_{l}\left(s\right)\cup W_{l}\left(t\right)}\left(X_{F^{\prime }}\right) \end{array}$$

where *W*_*l*_(*t*) = (*t* − *l*/2, *t* + *l*/2) is the time window around *t* with length *l*, $D_{W}\left(X_{F}\right)$ is the projection of the distribution process onto the features *F*, and $D_{W}\left(X_{F}|X_{F^{\prime }}\right)$ is the conditioning of $D_{W}\left(X_{F},X_{F^{\prime }}\right)$ on $D_{W}\left(X_{F^{\prime }}\right)$. The theoretical properties of the drift magnitude are analyzed by Hinder et al. (2021a). Notice that the drift magnitude on consecutive windows also forms the basis for the Shape Drift Detector (Hinder et al., 2021a).

To estimate the drift magnitude, Webb et al. (2017, 2018) use sliding windows (stage 1), and grid binning (stage 2) which are used to compute the total variation norm for different time points (stage 3). The results of this computation are directly presented to the user.

*ConceptExplorer* is a tool presented by Wang et al. (2020). It is designed for visual inspection of drift, in particular, in time-series data. The tool contains several analysis and visualization tools: a drift detection algorithm and an event-log-plot, an automatic extraction of concepts, a visualization, interaction, feature selection and relevance tools, and a cross-data source analysis. For drift detection and feature analysis, standard tools are used. The concept analysis is mainly performed by making use of a time-binned correlation matrix.

Pratt and Tschapek (2003) suggest using *brushed, parallel histograms* in order to visualize concept drift. The data distribution for each dimension is displayed using a histogram, correlations are marked by lines connecting the dimension-wise projections. The implementation presented by the authors enables user interaction by allowing for selecting subsets of points, e.g., parts of the histograms, for which more information is desired.

To visualize drift, the authors use sliding windows (stage 1) for which histograms are presented side-by-side (stages 2 & 3).

#### 4.3.2 Model-based drift explanations

The notion of model-based drift explanations was coined by Hinder et al. (2022a, 2023a). Simply put, the fundamental idea is that drift explanations are supposed to tell us why a drift detector raised an alarm. As stated by Hinder et al. (2024a) several approaches explicitly make use of machine learning models as a descriptor to detect the drift. In these cases, explaining why the model used by the drift detector obtained its results also provides us with information on why the drift detector raised an alarm or not.

In order to obtain sufficiently informative explanations requires a specific training scheme. As discussed before, for drift detection frequently detecting a single drifting feature is sufficient. Since this may be insufficient to provide a complete explanation, relying on drift localization or segmentation is a more reasonable approach.

**Figure 8:**
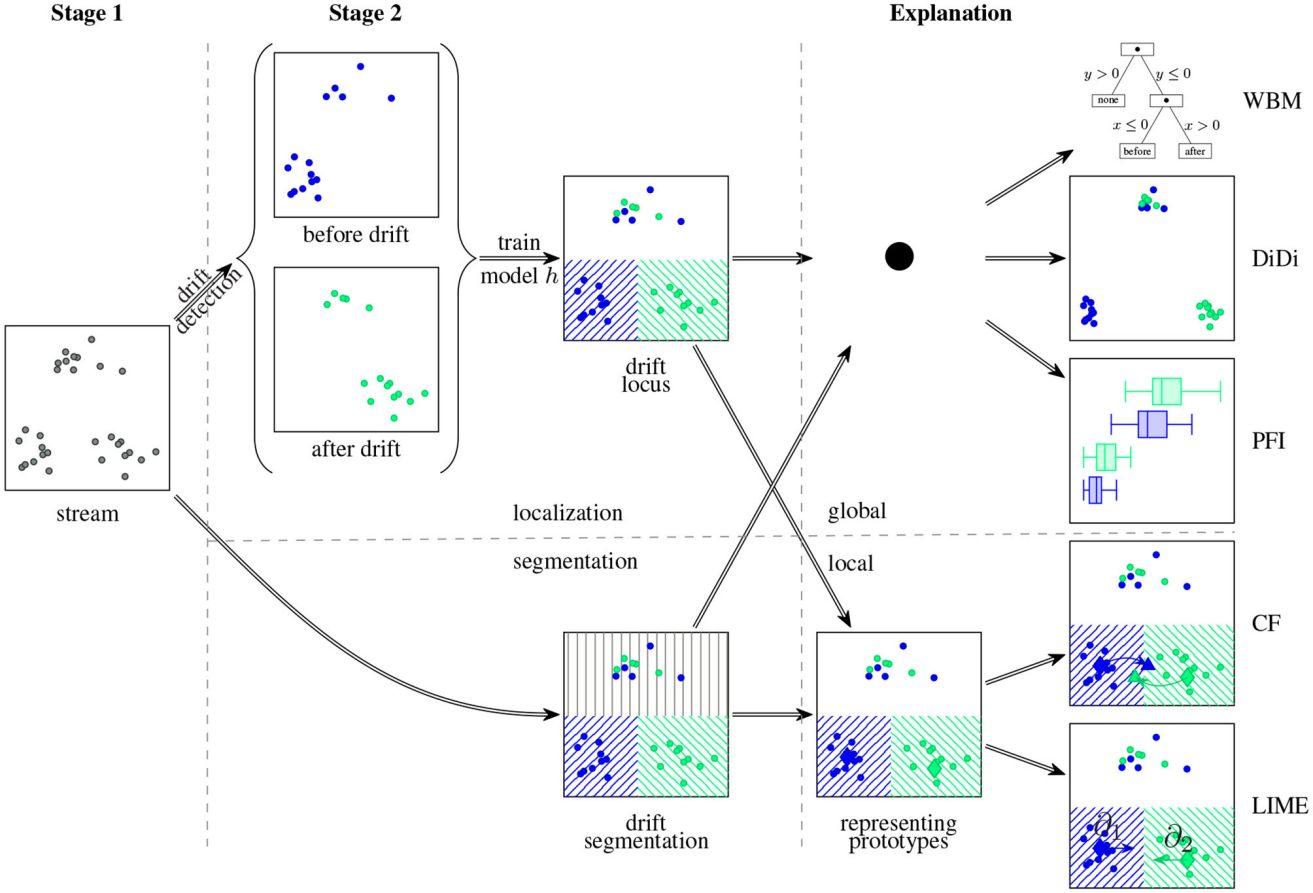
Model-based drift explanation strategy: In the first step a model for drift localization or drift segmentation is trained. Afterward, the obtained model is analyzed by means of an explanation method to gain insight into the drift.

Once a model is trained in an appropriate way we can obtain knowledge on the drift by analyzing it. This can be understood as follows: if the model contains all the information regarding the drift, we can analyze it as a proxy for the drift. This again fits into the three-staged scheme where the model serves as the descriptor: As visualized in Figure 8, first, data is acquired and a model describing the data is trained (stage 1&2). In the second step, the obtained model is explained by a suitable explanation approach (stage 3).

Explanations can be provided in several ways. We can thus choose the model and explanation that fit our problem best. In the original paper, the authors considered the non-exhaustive list of the following explanation methods (Hinder et al., 2023a):

- Linear models or decision trees belong to the class of *interpretable models* which can naturally be understood by humans (Du et al., 2019; Molnar, 2020). Yet, they usually suffer problems due to low complexity.
- More complex explanations are provided by *discriminative dimensionality reduction* which provides a global overview of the model behavior using model-enriched dimensionality reduction techniques (Venna et al., 2010; Schulz et al., 2020; Yang et al., 2020).
- *Global feature importance and relevance* techniques like permutation feature importance, feature importance, and Shapley-values offer feature-wise explanations (Shapley, 1951; Breiman, 2001; Nilsson et al., 2007). In contrast to other methods, those usually come with formal descriptions and guarantees on what they can and cannot do (Hinder and Hammer, 2023). Such are useful for various setups with semantic features, in particular sensor networks (Hinder et al., 2023a; Vaquet et al., 2024b).
- *Local feature importance* techniques like Saliency Maps (Simonyan et al., 2013) or Local Interpretable Model-agnostic Explanations (LIME) (Ribeiro et al., 2016) offer feature-wise analysis on a single instance basis. This can provide more information on the single instance and offers insights into the change of correlations, however, it also requires finding samples that are relevant enough to provide additional insight if analyzed. There are technique for finding such samples based on informed clustering (Hinder et al., 2023a).
- *Contrasting explanations and counterfactuals* offer explanations in terms of contrasting sample pairs (Looveren and Klaise, 2019; Molnar, 2020; Yang et al., 2021). In contrast to local feature-wise explanations, those do not only show which features are affected but also how they are affected. Thus, the user is directly confronted with the effect of the drift in exemplary cases. The drawbacks of this approach are that it only works well with abrupt drift, is computationally expensive, and there are usually no guarantees that valid explanations are found.

A further advantage of model-based explanations is that the connection of drift-related problems like drift detection and localization to explanation and analysis techniques can also be used to increase performance. For example, this connection is not only used to transfer ideas of feature relevance theory in order to obtain drift explanations but also to perform feature selection for drift detection which resulted in significant increases in accuracy (Hinder and Hammer, 2023).

There do exist works prior to Hinder et al. (2023a) that make use of a similar schemes. However, those approaches are hand-tailored for a specific setup rather than a general framework. Yang et al. (2021) use a combination of an auto-encoder and a distance-based outlier detection in the latent space for drift detection. Drift explanations are provided by counterfactuals of the outlier detector. Yang et al. (2020) detect drift using a Gaussian mixture model in a loss-based fashion. Then, the authors use a discriminative version of t-SNE to create an embedding.

### 4.4 Conclusion and guidelines

Focusing on drift explanations, we identified another research gap, as much of the work in this area is still very basic. Much more work is needed to provide user-friendly explanations across different domains and settings. Additionally, evaluations in the form of user studies will be required to evaluate future approaches. Regarding the discussed methodologies, model-based explanations seem the most promising as the framework is very flexible combining model-based localization and segmentation methods with a range of established explanation schemes. The latter can be chosen to fit the real-world scenario that needs to be targeted.

## 5 Conclusion

In this work, we provided a definition and categorization of drift localization in an unsupervised setting. Furthermore, we categorized state-of-the-art approaches and analyzed them based on a four-staged general scheme we proposed. In addition, we briefly considered drift explanations and showcased some works targeting this task.

Next to providing an overview of existing work, we analyzed the different underlying strategies to contribute guidelines on how to choose methodologies based on the attributes of the setup and the expected drift mechanism. Finally, we found that more research focusing on the localization and explanation tasks is needed.

## Data availability statement

The datasets presented in this study can be found in online repositories. The names of the repository/repositories and accession number(s) can be found at: https://github.com/FabianHinder/One-or-Two-Things-We-Know-about-Concept-Drift.

## Author contributions

FH: Conceptualization, Formal analysis, Investigation, Methodology, Visualization, Writing – original draft, Writing – review & editing. VV: Conceptualization, Formal analysis, Investigation, Methodology, Visualization, Writing – original draft, Writing – review & editing. BH: Conceptualization, Funding acquisition, Supervision, Writing – review & editing.

## Footnotes

^1^All considerations below also work in a very similar way for more general measure spaces. However, as some formal issues may arise from that, we will stick with this far simpler special case for the sake of clarity. This also holds for the restriction of *P*_*T*_ to be a probability measure which is usually specified by term *post hoc*.

^2^Both existence and uniqueness of D are assured by the Fubini-Tonelli theorem.

^3^The experimental code can be found at https://github.com/FabianHinder/One-or-Two-Things-We-Know-about-Concept-Drift
